# Association of Radiation-Induced Acute Esophagitis With Dosimetric Parameters of Oesophagus in Breast Carcinoma Patients Receiving Supraclavicular Nodal Irradiation

**DOI:** 10.7759/cureus.60778

**Published:** 2024-05-21

**Authors:** Sadia Sharmin, Rokaya Sultana, Nazir Uddin Mollah, Mamun O Rasheed, Afsana Sharmin Anika, Md Rassell

**Affiliations:** 1 Department of Clinical Oncology, Bangabandhu Sheikh Mujib Medical University, Dhaka, BGD; 2 Department of Surgical Oncology, Bangabandhu Sheikh Mujib Medical University, Dhaka, BGD

**Keywords:** association, supraclavicular nodal irradiation, breast carcinoma, oesophagus, radiation-induced acute esophagitis

## Abstract

Introduction: We conducted this investigation to ascertain the dosimetric properties such as the mean and maximum radiation dosage during radiotherapy as well as the extent of radiation exposure to the esophagus. These factors can potentially impact the development of esophagitis in breast cancer patients undergoing supraclavicular radiation.

Methodology: From January to June 2023, an observational study was conducted at Bangabandhu Sheikh Mujib Medical University in Bangladesh. The patients received radiation therapy (40.05 Gy in 15 parts) to the chest wall and supraclavicular node for three weeks. We were able to guess the following from the dose volume histogram (DVH) data: the length of the esophagus in the treatment area (i.e., the size of the esophagus that was visible on the planning CT scan), the maximum dose (D_max_), the mean dose (D_mean_), and the volume of the 10Gy (V_10Gy_) and 20Gy (V_20Gy_) doses that were given to the esophagus. During radiotherapy, patients were checked on once a week, and the radiotherapy oncology group was used to evaluate and grade esophagitis

Results: Patients with left-sided breast cancer showed a higher D_mean_, D_max_, and length of the esophagus compared to those with right-sided breast cancer. Specifically, the D_mean_ was 6.7 (±2.1) Gy, the D_max_ was 39.2 (±1.5) Gy, and the length of the esophagus was 6.1 (±1.2) Gy. Patients with left breast cancer had elevated V_10Gy_ and V_20Gy_ values for the esophagus, but the difference was not statistically significant. The incidence of V_10Gy_ for right-sided breast cancer and left-sided breast cancer was 4.2% (±2.6%) and 19.8% (±9.2%), respectively. The V_20Gy_ was 2.4% (±0.9%) for right-sided breast cancer and 13.09% (±5.0%) for left-sided breast cancer

Conclusion: In conclusion, there is a strong association between the mean oesophageal dose and radiation to the left supraclavicular region following surgery in women with breast cancer and acute esophagitis. We can reduce esophageal toxicity by prescribing dose restrictions and performing precise delineation of the esophagus.

## Introduction

Breast carcinoma is the predominant kind of cancer that impacts women globally, especially those in South Asia [1,2]. Globally, it is the most prevalent type of cancer, accounting for an estimated 19,292,789 reported cases and 9,958,133 fatalities in 2020 [3]. Radiotherapy is a crucial component in the comprehensive treatment of breast cancer patients, spanning from the initial stages to those that have spread locally or to other parts of the body [4]. Furthermore, using radiation therapy (RT) to target the lymph node area in breast cancer patients with confirmed positive lymph nodes lowers both the overall death rate and the chance of the cancer coming back [4]. Prospective studies and meta-analyses have shown that adding radiotherapy to a mastectomy improves cancer treatment in the local and regional areas and raises the chance of survival for breast cancer patients who have positive lymph nodes after a mastectomy [5,6]. The American Society of Clinical Oncology (ASCO) initially advised supraclavicular radiation for all patients with four or more affected lymph nodes [7]. The revised ASCO guidelines recommend the utilization of supraclavicular RT, even in cases where there are only one to three affected lymph nodes [8].

Nevertheless, there is a belief that RT targeting the lymph nodes in the supraclavicular fossa (SCF) can induce inflammation in the esophagus, a condition referred to as esophagitis. The esophagus is located adjacent to the SCF node, predominantly on the left side of the cervical spine. Three morphological divisions divide the organism: cervical, thoracic, and abdominal. The esophagus originates at the cricoid cartilage's inferior border. The esophagus is primarily oriented vertically, with two noticeable bends present. Starting from the midline, it first slopes towards the left, extending down to the base of the neck, and then gradually returns to the midline at the level of the fifth thoracic vertebrae before finally veering to the left. It progresses by causing a tear in the esophagus around the diaphragm and stomach [9].

As a result, administering radiotherapy to the supraclavicular lymph nodes may expose a significant portion of the esophagus to radiation, which could potentially heighten the likelihood of developing acute radiation esophagitis (RE). Yaney et al. found that 16.2% of patients (86 out of 531) who underwent RT for regional lymph nodes experienced grade 2 esophagitis. As a result of the implementation of intensity-modulated radiation treatment (IMRT), esophagitis became more common [10]. Researchers have extensively studied esophagitis in the context of RT targeting the lungs, head, and neck [11-17]. However, even in these investigations, it was not feasible to ascertain the specific dosage at which esophagitis was prone. However, the esophagus, including the supraclavicular lymph nodes, received a mean radiation dose of 11.4 Gy [18]. 

There is a scarcity of data that establishes a connection between the measurement of radiation dose in the esophagus and the occurrence of esophagitis in breast cancer patients. Therefore, implementing dose restrictions can effectively decrease radiation exposure to the esophagus, potentially enhancing the overall quality of life. West et al. conducted a study that examined the parameters associated with acute esophageal toxicity in individuals who underwent conventional fractionation radiation (50 Gy in 25 fractions). As a result, we conducted this investigation to ascertain the dosimetric properties, such as the mean and maximum radiation dosage during radiotherapy, as well as the extent of radiation exposure to the esophagus. These factors can potentially impact the development of esophagitis in breast cancer patients undergoing supraclavicular radiation.

## Materials and methods

This was an observational study conducted at Bangabandhu Sheikh Mujib Medical University, Dhaka, Bangladesh, from January to June 2023. The Institutional Ethics Committee of Bangabandhu Sheikh Mujib Medical University provided ethical clearance for the project (approval number: IEC/BSMMU/APPROVAL/BCD-10112/12/2022). Before data collection, informed consent was obtained from each patient.

This investigation enrolled and analyzed 23 patients with pathologically positive axillary lymph nodes and histologically proven breast cancer. All patients having a history of prior radiation, recurring disease, esophageal disease, positive supraclavicular and internal mammary nodes, distant metastases, instances involving only the chest wall, and treatment plans for individuals with an enlarged thyroid were excluded from the research.

Pre-treatment medical history was documented along with the outcomes of the general physical and local examinations. The patients received RT (40.05 Gy in 15 parts) to the chest wall and supraclavicular node for three weeks. We utilized the breast contouring criteria developed by the Radiation Therapy Oncology Group (RTOG) to determine the clinical target volume and organs at risk [19]. We were able to estimate the following from the Dose Volume Histogram (DVH) data: the length of the esophagus in the treatment area (i.e., the size of the esophagus that was visible on the planning CT scan), the maximum dose (D_max_), the mean dose (D_mean_), and the volume of the 10Gy (V_10Gy_) and 20Gy (V_20Gy_) doses that were given to the esophagus. During radiotherapy, patients were checked on once a week, and the radiotherapy oncology group was used to evaluate and grade esophagitis [19].

Statistical studies were conducted using IBM SPSS Statistics for Windows, Version 23.0 (Released 2015; IBM Corp., Armonk, New York, United States). We used the mean and standard deviation (SD) to represent continuous variables and reported frequency and percentage for categorical variables. Student's t-test was used to compare groups with constant parameters. The Chi-Square test evaluated distinct criteria. The findings were deemed statistically significant if the p-value was below 0.05.

## Results

A total of 23 breast cancer patients received RT to the chest wall and supraclavicular nodes. We examined the dosimetric values, which include the D_max_, D_mean_, V_10Gy_, and V_20Gy_, and the length of the esophagus inside the treatment area. The age range was 28-66 years. The patients' average age at diagnosis was 44.6 ± 10 years. Most patients (56.5%) exhibited the illness in their left breast and 69.5% of the patients were classified as Stage II (Table 1). 

**Table 1:** Distribution of patients according to the demographic and clinical characteristics

Parameters	Values
Age in years, mean ± SD)	44.6 ±10.14
Laterality of the disease, n (%)	
Left	13 (56.5%)
Right	10 (43.4%)
Stage of disease, n (%)	
II	16 (69.5%)
III	7 (30.4%)

The D_mean_ and D_max_ received by the esophagus were 4.7 ± 2.8 Gy and 35.05 ± 7.52 Gy, respectively. The length of the esophagus inside the treatment area was 5.3 ± 1.4 cm. Furthermore, the esophagus was exposed to a radiation dose of 10 Gy and 20 Gy, resulting in a volume of 13.06 (±10.05%) and 8.4 (±6.5%), respectively (Table 2).

**Table 2:** Dosimetric parameters D_mean_: mean dose; D_max_: maximum dose

Variable	Mean+SD
D_mean_(Gy)	4.72±0.8
D_max_(Gy)	35.05±7.52
Length of esophagus inside the radiation field (cm)	5.3±1.4
V_10Gy _(%)	13.06±10.5
V_20Gy_ (%)	8.4±6.5

Patients with left-sided breast cancer showed a higher D_mean_, D_max_, and length of the esophagus compared to those with right-sided breast cancer. Specifically, the D_mean_ was 6.7 (±2.1) Gy, the D_max_ was 39.2 (±1.5) Gy, and the length of the esophagus was 6.1 (±1.2) Gy. Patients with left breast cancer had elevated V_10Gy_ and V_20Gy_ values for the esophagus, but the difference was not statistically significant. The incidence of V_10Gy_ for right-sided breast cancer and left-sided breast cancer was 4.2% (±2.6%) and 19.8% (±9.2%), respectively. The V_20Gy_ was 2.4% (±0.9%) for right-sided breast cancer and 13.09% (±5.0%) for left-sided breast cancer (Table 3).

**Table 3:** Correlation of dosimetric parameters with the laterality of disease D_^mean^_: mean dose; D_max_: maximum dose

Variable	Laterality of breast treated	Mean±SD	P-value
D_mean_(Gy)	Left	6.7 ±2.1	0.4
	Right	2.1 ±0.7	
D_max_(Gy)	Left	39.2 ±1.5	0.4
	Right	29.6 ±8.7	
Length of esophagus (cm)	Left	6.1 ±1.2	0.07
	Right	2.1 ±0.7	
V_10Gy _(%)	Left	19.8 ±9.2	0.34
	Right	4.2 ±2.6	
V_20Gy _(%)	Left	13.09+5.0	0.4
	Right	2.4+0.9	

Exposure to a dosage of more than 5 Gy led to a greater severity of esophagitis. The average radiation exposure to the esophagus was considerably greater in five out of 38 individuals (13%) (p = 0.02). No patients experienced grade 2 esophagitis when the average radiation dosage was below 5 gray. The study found that individuals with left-sided breast cancer had a more significant incidence of grade 2 esophagitis compared to those with right-sided breast cancer. The incidence was 39.1% for left-sided breast cancer and 8.6% for right-sided breast cancer (P = 0.02). It wasn't clear whether esophagitis was linked to the length of the treated esophagus (P = 0.06), the highest dose given to the esophagus (P = 0.73), or the amount of radiation given at 10 Gy (P = 0.34) or 20 Gy (P = 0.40) (Table 4).

**Table 4:** Corelation of dosimetric parameters and laterality of disease with the frequency of esophagitis D_mean_: mean dose; D_max_: maximum dose

Variable	Grade 1		Grade 2		Total		p-value
D_mean_(Gy)	n	%	n	%	n	%	
< 5 Gray	11	56.50	0	0.00	11	47.80	0.02
>5 Gray	7	30.40	5	13.00	12	52.10	
D_max_(Gy)							
<36 Gray	5	21.70	1	4.30	6	26.00	0.73
>36 Gray	14	60.80	3	13.00	17	73.90	
Length of esophagus (cm)							
<6 cm	7	30.40	2	8.60	9	39.10	0.06
>6 cm	5	21.70	9	39.10	14	60.80	
Laterality of disease							
Left	4	17.39	9	39.10	13	56.50	0.02
Right	8	34.70	2	8.60	10	43.40	
V_10Gy _(%)	15	65.20	8	34.70	23	100.00	0.34
V_20Gy _(%)	12	52.10	11	47.80	23	100.00	0.4

**Figure 1:**
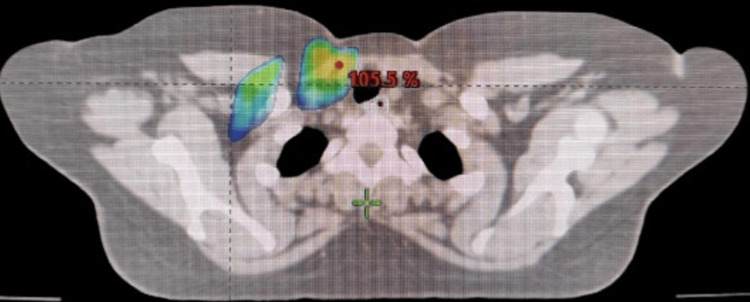
Axial slice of right breast of a patient

The axial slice shows the intersection of the 95% (38.04 Gy) line with the esophagus, with the treatment fields for a patient with right breast cancer stacked on top of each other. The delivered amount was 40.05 Gy, divided into 15 pieces (Figure 1). 

**Figure 2:**
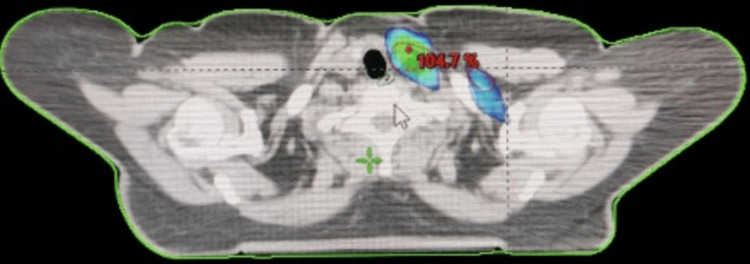
Axial slice of left breast of a patient

Additionally, Figure 2 shows the treatment fields positioned above for a patient with a left breast cancer diagnosis. The prescribed dose was 40.05 Gy, delivered in 15 fractions.

## Discussion

RT for breast cancer patients increases the risk of developing esophagitis as the amount of radiation exposure to the esophagus increases. The presence of supraclavicular lymph nodes was the primary factor affecting the mean and maximum esophageal dosage. Individuals with esophagitis had a significantly higher average dose to the esophagus. This study exposed the esophagus to a D_mean_ of 4.72 Gy and a D_max_ of 35.05 Gy. These findings are consistent with a prior study by Wang and colleagues [20]. In a study by DeSelm et al., the multivariate analysis revealed a strong correlation between esophagitis and the D^mean^ [21]. Meanwhile, Wijsman research discovered the D_mean_ as a reliable indicator of acute esophagitis [22]. According to our study, patients with breast cancer receiving RT above the collarbone can predict the likelihood of developing esophagitis with great accuracy using the esophageal D_mean_.

Our data, in line with the West et al. study, show no clear link between the highest dose the esophagus receives, its length within the treatment area, or the number of times it becomes esophageal toxic [9]. The left SCF is closer to the esophagus and may overlap it because of the esophagus's slight deviation to the left during its descent. In contrast to the right-sided SCF, this may lead to higher radiation dosages reaching the esophagus [23]. During our study, we found that the left side of the esophagus had higher D_max_ and D_mean_ values (6.7±2.1 Gy and 39.2±1.5 Gy, respectively) than the right side. These variations, however, lacked statistical significance.

Compared to patients with right-sided breast cancer (8.6%), patients with left-sided breast cancer had a greater incidence of grade 2 esophagitis (39.1%). A statistically significant difference is indicated by the p-value of 0.02. This occurrence could be explained by the left-sided position of the esophagus relative to the SCF. Bhaskaran et al.'s study reveals that left-sided breast cancer exposes the esophagus to more radiation than right-sided breast cancer, which might cause an imbalance in the radiation dose that these two regions receive [23]. If someone has regular esophageal shaping, the right amount of radiation exposure, close patient monitoring during RT for esophageal inflammation, and good treatment process management [24], it is less likely that they will develop esophagitis. Because the esophagus is so close to field boundaries, even slight changes to field limits or angles can significantly impact the esophagus [25]. Our study employed the RTOG atlas to define the clinical target volume and organs at risk, including the esophagus. RE grade ≥1 was much more likely to happen in patients who used the RTOG atlas for delineation than in patients who used the ESTRO atlas (60.2% vs. 42.5%; P =0.006) [26]. The RTOG atlas moves the lymph nodes above the clavicle, closer to the head. As a result, the esophagus is closer to the RT field for regional nodal irradiation [27,28]. On the inner side of the carotid artery, there are no nodal recurrences. Still, there are several nodal recurrences in the area between the internal jugular vein and the carotid artery. According to the findings, the carotid artery's inner edge may serve as the supraclavicular region's innermost boundary, limiting the quantity of radiation that enters the esophagus [29]. Our study was one of the few that examined the variables influencing the radiation dose required for regional nodal irradiation during hypofractionated breast cancer treatment.

Limitations

There were some limitations to this investigation. Variations in the esophageal boundary may significantly affect the incidence of esophagitis. We did not include the assessment of chronic toxicity, particularly dysphagia and esophageal stricture. Our sample size was constrained. Moreover, we need more prospective studies with many patients to determine the correlation between esophageal toxicity and variables such as D_max_, D_mean_, V_10Gy_, V_20Gy_, and esophageal length. 

## Conclusions

Our study revealed that esophagitis was more common in breast cancer patients who had received supraclavicular nodal irradiation and was related to certain dosimetric factors, such as the average dose given to the esophagus (D_mean_) and tumor location. Individuals exposed to an average esophageal dosage exceeding 5 Gy were more likely to develop Grade 2 esophagitis. Even so, there was no statistically significant link between esophagitis and other things, such as the length of the esophagus near the treatment site, the highest dose given to the esophagus, or the amounts of radiation at 10 Gy and 20 Gy. These results show that it is essential to carefully look at dosimetric parameters when planning RT for breast cancer patients to lower their risk of esophagitis.
